# Replacement of *Leishmania (Leishmania) infantum* Populations in an Endemic Focus of Visceral Leishmaniasis in Brazil

**DOI:** 10.3389/fcimb.2022.900084

**Published:** 2022-06-24

**Authors:** Hugo O. Valdivia, Bruno M. Roatt, Rodrigo de Paula Baptista, Jennifer Ottino, Anderson Coqueiro-dos-Santos, Mandy J. Sanders, Alexandre B. Reis, James A. Cotton, Daniella C. Bartholomeu

**Affiliations:** ^1^Departamento de Parasitologia, Instituto de Ciências Biológicas, Universidade Federal de Minas Gerais, Belo Horizonte, Brazil; ^2^U.S. Naval Medical Research Unit Six, Department of Parasitology, Lima, Peru; ^3^Núcleo de Pesquisas em Ciências Biológicas/NUPEB, Universidade Federal de Ouro Preto, Ouro Preto, Brazil; ^4^CTEGD- Center for Tropical and Emerging Global Diseases and IOB - Institute of Bioinformatics, University of Georgia, Athens, United States; ^5^Houston Methodist Research Institute, Houston, United States; ^6^Wellcome Sanger Institute, Cambridge, United Kingdom; ^7^Institute of Biodiversity, Animal Health and Comparative Medicine, Wellcome Centre for Integrative Parasitology, University of Glasgow, Glasgow, United Kingdom

**Keywords:** *L. infantum*, genomics, genetic variability, Brazil, population replacement

## Abstract

Visceral leishmaniasis is an important global health problem with an estimated of 50,000 to 90,000 new cases per year. VL is the most serious form of leishmaniasis as it can be fatal in 95% of the cases if it remains untreated. VL is a particularly acute problem in Brazil which contributed with 97% of all cases reported in 2020 in the Americas. In this country, VL affects mainly the poorest people in both urban and rural areas and continues to have a high mortality rate estimated around 8.15%. Here, we performed a temporal parasite population study using whole genome sequence data from a set of 34 canine isolates sampled in 2008, 2012 and 2015 from a re-emergent focus in Southeastern Brazil. Our study found the presence of two distinct sexual subpopulations that corresponded to two isolation periods. These subpopulations diverged hundreds of years ago with no apparent gene flow between them suggesting a process of rapid replacement during a two-year period. Sequence comparisons and analysis of nucleotide diversity also showed evidence of balancing selection acting on transport-related genes and antigenic families. To our knowledge this is the first population genomic study showing a turn-over of parasite populations in an endemic region for leishmaniasis. The complexity and rapid adaptability of these parasites pose new challenges to control activities and demand more integrated approaches to understand this disease in New World foci.

## Introduction

Leishmaniasis is a complex parasitic disease with diverse clinical manifestations that can range from self-curing cutaneous lesions to deadly visceral compromise. This disease is caused by digenetic protozoa from the genus *Leishmania* that are transmitted to mammalian hosts by the bite of infected sand flies: *Lutzomyia* in the New World and *Phlebotomus* in the Old World. An important feature of the leishmaniasis is the diverse spectrum of clinical manifestations associated with the 20 human pathogenic *Leishmania* species. These clinical features have been classified into tegumentary leishmaniasis (TL), mucosal leishmaniasis (ML) and visceral leishmaniasis (VL) (Murray et al., 2005; David and Craft, 2009). VL stands out as the most important form of leishmaniasis due to its potential to cause death, although the range of symptoms associated with VL are also diverse and can vary from subclinical to fully established infection leading to fever, general weakness, weight loss, multi-organ failure and death (Murray et al., 2005; Banuls et al., 2007).

Leishmaniasis is present in more than 98 countries, with up to 1.5 new million cases per year and putting at risk 350 million people (Murray et al., 2005; Alvar et al., 2012). Surveillance data indicates that 0.2 to 0.4 million cases each year are due to VL and up to 90% of these cases occur in India, Bangladesh, Sudan, South Sudan, Brazil and Ethiopia (Desjeux, 2004; Alvar et al., 2012) constituting an important public health problem in these countries.

The use of high-throughput sequencing technologies has contributed to a better understanding of the biology and mechanisms associated with parasitism in *Leishmania*. Genomic studies on the genus have resulted in the genomic characterization of several *Leishmania* species (Ivens et al., 2005; Peacock et al., 2007; Aslett et al., 2010; Real et al., 2013) and the assessment of parasite population structure in endemic areas in the Old World (Downing et al., 2011; Rogers et al., 2014; Franssen et al., 2020). These endeavors have revealed the genomic organization of *Leishmania* into bidirectional gene clusters (Ivens et al., 2005), a high level of synteny across species (Peacock et al., 2007) and the influence of aneuploidy and gene duplication as a mechanism to increase gene expression in the absence of a regulatory transcriptional mechanism (Rogers et al., 2011).

In the New World, genomic studies are starting to be used for in depth characterization of different aspects of *Leishmania* biology such as drug resistance, dynamics of parasite populations and evolutionary history (Teixeira et al., 2017; Carnielli et al., 2018; Van Den Broeck et al., 2020; Schwabl et al., 2021). Results from these studies have highlighted important gaps in knowledge and the need to extend the use of genomics to characterize other endemic areas.

Here, we used Illumina sequencing to generate genome-wide data for 34 canine *L. (L.) infantum* isolates from the city of Governador Valadares which is a reemerging VL focus since 2008 after the interruption of control activities (Barata et al., 2013). Analysis of single nucleotide polymorphisms allowed us to reveal the presence of two distinct subpopulations and a rapid replacement of one population by the other during a two-year period. Our analyses further suggest that these two subpopulations diverged more than 300 years ago and there was no apparent genetic exchange between them since that time. Neutrality tests allowed us to identify regions under balancing selection that code for important antigenic families that mediate host parasite interactions like amastins, GP63, cathepsin-L among others. This study presents evidence of the high adaptability and dynamism of the *L. (L) infantum* population in the city of Governador Valadares with potential implications to other endemic foci. To our knowledge, it is the first time that turn-over of parasite populations within a single *Leishmania* focus has been described.

## Material and Methods

### Study Site, Sample Collection and Parasite Isolates

**Figure 1:**
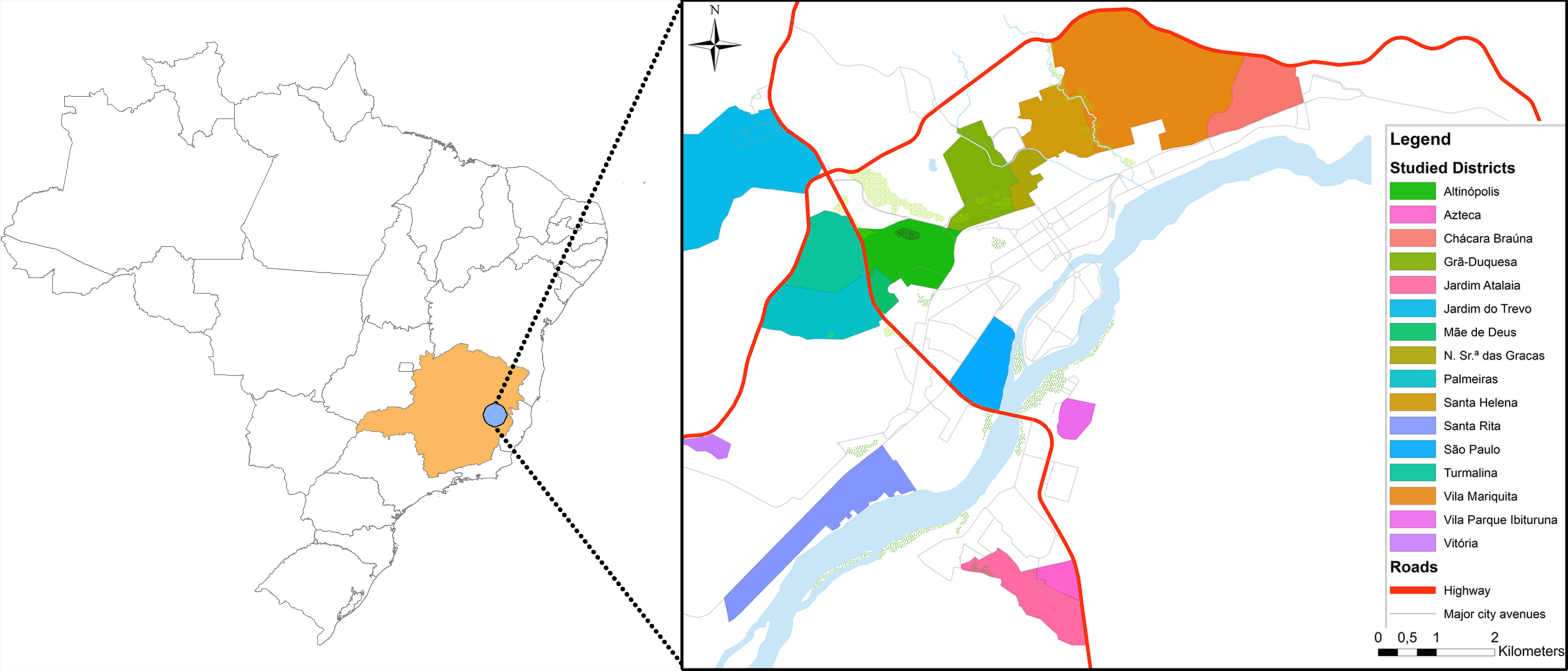
Map of the city of Governador Valadares. On the left the inset shows the location of the Minas Gerais State in Brazil in orange and the city of Governador Valadares City as a blue point. On the right we show a city map with the neighborhoods where isolates were collected.

Samples were collected in 2008 (n=9), 2012 (n=5) and 2015 (n=20) from domestic dogs with clinical symptoms of leishmaniasis from the city of Governador Valadares in the State of State of Minas Gerais in Brazil (**Figure 1**). This city is located at the bank of the Doce River at 455 meters above sea level and presents a tropical sub-humid climate (Kottek et al., 2006). This city is considered a re-emerging focus of tegumentary and visceral leishmaniasis with more than 127 human cases reported between 2007 and 2013 (Saúde and Saúde, 2007) and approximately 30% of domestic dogs positive for leishmaniasis by serology based on previous studies (Barata et al., 2013) and surveillance data obtained from the city of Governador Valadares Visceral Leishmaniasis control program (**Supplementary Figure 1**).

All parasite isolates were obtained from bone marrow aspirates and used for *in vitro* culture. Parasites were cultivated in Schneider culture medium supplemented with 10% fetal bovine serum and 1% penicillin and streptomycin for up to three passages limited to one month of *in vitro* culturing. All experimental procedures were approved by the Committees of Ethics in Animal Experimentation of the Universidade Federal de Ouro Preto (protocol number 083/2007) and were conducted according to the guidelines set by the Brazilian Animal Experimental College (COBEA), Federal Law number 11794.

Genomic DNA was extracted from ≈10^9^ promastigotes using the DNeasy Blood and Tissue Kit (Qiagen) using the manufacturers protocol.

### Sequencing

Genomic DNA was sheared into 400–600-base pair fragments by focused ultrasonication (Covaris Adaptive Focused Acoustics technology -AFA Inc., Woburn, USA) and standard Illumina libraries were prepared. These libraries were used to produce 100 base-pair paired-end reads on the HiSeq 2000 v3 according to the manufacturer’s standard sequencing protocol. The reference genome of *L. (L.) infantum* JPCM5 (Peacock et al., 2007) was downloaded from the Tritryp database v.46 (http://tritrypdb.org/) for subsequent bioinformatics analyses. Sequencing reads from this project was deposited in the European Nucleotide Archive (ENA) and the accession numbers are provided in the **Supplementary Table 1**.

### Filtering and Mapping

Illumina reads had their qualities checked using FASTQC and those reads with lower quality regions were trimmed using Trimmomatic 0.36 (Bolger et al., 2014) with minimum base quality cutoff of 30, leading and trailing base qualities of 28, minimum per base average quality of 20 and a minimum read length of 70bp. Filtered reads were then mapped onto the *L. (L.) infantum* strain JPCM5 (TrytripDB v.46) reference genome using BWA-MEM v0.7.17 (Li and Durbin, 2009). The alignment then parsed using Picard toolkit v2.16.0 to mark duplicates and remove unmapped reads for copy number analysis and variant calling.

### Population Structure Analysis

Variants (SNPs and InDels) among the isolates were called using the recommended parameters of GATKv3.8 (Mckenna et al., 2010). All variants were called, for each sample, using the haplotypecaller module for ploidy equals to 2, a minimum phred-scaled confidence threshold of 30 for calling (stand_call_conf) and outputted as a genomic vcf (GVCF), which records all genomic positions in the genome including regions where no variant was detected. All GVCFs raw variants were then filtered selecting sites with minimum raw depth coverage (DP) of 10, Root Mean Square mapping quality (MQ) of 40, quality by depth (QD) greater than 1.5, haplotype score greater than 13 and a FisherStrand bias score (FS) lower than 60 to avoid false positive calls.

Multiallelic variants were tagged using a custom bash script, for later filtration and all GVCFs were combined using into a unique variant file using the GATK GenotypeGVCF module. The combined GVCF was then converted to fasta by using a custom bash script that generate a consensus variant sequence for each sample including both SNPs and InDels and remove all tagged multialletic variants from the analysis.

The combined GVCF with all isolates was also used for population analyses using ADMIXTURE v1.3.0 (Alexander et al., 2009). The GVCF was first converted to a plink format using VCFtools v0.1.15 (Danecek et al., 2011) and a bed file was generated using PLINK v.1.9b (Purcell et al., 2007). The cross-validation flag (–cv) was used to calculate the statistical likelihood of each K-value. Lower cv values represents best K-values result interpretation and visualization.

### Phylogenetic Analysis

Phylogenetic analysis of the concatenated dataset was based on maximum likelihood under a separate genotype-aware phylogenetic Jukes-Cantor (GTJC) model for each chromosome and branch length scale allowed to vary between chromosomes, using raxml-ng v0.8.1 (Stamatakis, 2014). An initial run was performed using default settings, with tree search starting from 10 random trees and 10 random step-wise addition parsimony trees, followed by fine optimization of branch lengths and model parameters using –precision 8 –lh-epsilon 0.001. Support for partitions on the tree was assessed with 200 bootstrap replicates.

### Linkage Disequilibrium Analysis

R^2^ between pairs of SNP variants was calculated using the geno-r2 command in VCFtools v0.1.16 for all pairs of variants up to 500,000 bp apart, and reporting values for SNPs with a minimum R^2^ of 0.001, between-chromosome values were calculated using the –interchrom-geno-r2 command.

### Chromosome and Gene Copy Number Analysis

To estimate haploid chromosome copy number, we normalized the median read depth for each chromosome by the median read depth of the whole genome using an in-house Perl script and BEDtools (Quinlan, 2014). Data were plotted on a heatmap graph generated by using RStudio v1.1.453. Gene copy number variations were assessed by a single-copy gene normalization method as previously described (Valdivia et al., 2015).

### Allele Frequency Distribution

To evaluate ploidy levels we estimated the allele frequencies for all isolates using SAMtools (Williams et al., 2009). Briefly, for each heterozygous site having two variants, we estimated the number of reads showing the alternate and reference bases. Counts for each chromosome were grouped in bins from 0.01 to 1.0 and we plotted the proportion of sites in each frequency bin. Heterozygous sites on disomic chromosomes will have a tendency towards frequencies close to 0.5, while those on trisomic chromosomes will show frequencies of 1/3 or 2/3 and those on tetrasomic chromosomes can show peaks at 1/4, 1/2 and 3/4.

### Divergence Time Estimation

Inferred sequences based on variant calls for each isolate were aligned against the *L. (L.) infantum* JCPM5 reference with MAFFT v7.407 and poorly aligned regions were removed with trimAL v1.4.1 (Capella-Gutierrez et al., 2009). The alignment was then run under a coalescent model on BEAST v2.5 (Bouckaert et al., 2019) under the strict and relaxed log normal clocks. Times of divergence were obtained by converging three independent Markov Chain Monte Carlo (MCMC) runs of 50 million generations. The date of the root node (representing divergence between JPCM5 and samples from the city of Governador Valadares) was constrained using a calibration chosen to approximate divergence dates already estimated between Old World and New World *L (L.). infantum* (500 ya, SD 200 years) and between *L. (L.) donovani* and *L (L.). infantum* (0.95 Mya, SD 0.1 Mya) under Yule speciation model (Leblois et al., 2011). TreeAnnotator was used to summarize the sample of trees produced by BEAST onto a single tree using a Burn-in of 20% of the samples. Tree topology and summary information was visualized using Figtree.

### Climate Data Analysis

Daily precipitation and temperature data from 2008-2014 were obtained from the Instituto Nacional de Meteorologia (INMET). Mean daily values were obtained and used for cumulative and 3d surface plots on R. To evaluate if there were substantial variation of precipitation through these years, we employed TREND for statistical analysis of annual and seasonal rainfall time series using the Worsley likelihood ratio test.

## Results

### Mapping and Variant Calling Statistics

Genome sequencing resulted in an average of 12,373,596 reads per sample with 92% of them mapping into the *L. (L.) infantum* reference genome resulting in an average read depth of 39x and 99% of the genome covered by more than 5 reads. Variant calling resulted in 338 SNPs and 13 INDELs called per sample in average.

### Phylogenetic and Clustering Analysis Show the Presence of Two Distinct Sub-Populations in the City of Governador Valadares

**Figure 2:**
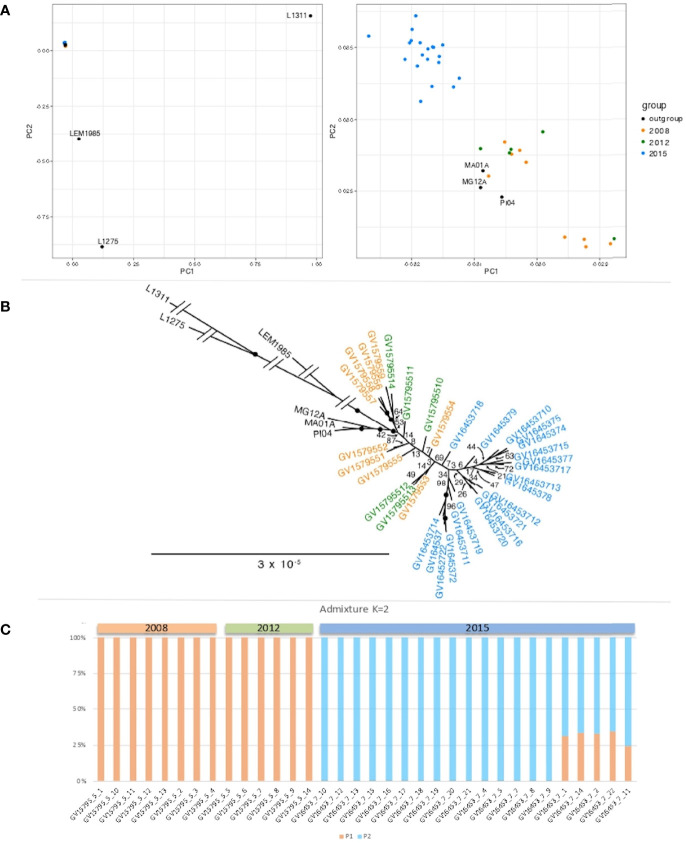
Genomic relationship between Governador Valadares *L. (L.) infantum* isolates. **(A)** Principal component analysis (PCA) based on genome-wide SNP variations. Left panel includes Brazilian and Old World isolates (Israel: LRC-L1275, France: LEM1985 and Uzbekistan: LRC-L1311. Right panel is a zoom-in of the left panel showing only the Brazilian isolates (colored dots are Governador Valadares isolates and black dots are isolates from other Brazilian regions: MG12A, Montes Claros from Minas Gerais state; MA01A, Paraibano from Maranhão state and PI04, Valença from Piauí state. The first two PCA axes represent 53.12% and 13.58% of the variance, for a total of 66.7%. **(B)** Maximum likelihood phylogenetic tree showing two Governador Valadares isolate clades. **(C)** Population structure results confirming the presence of two distinct *L. (L.) infantum* subpopulations in the city of Governador Valadares, orange bars represent ancestral population 1, whereas blue bars represent population 2.

We assessed the relatedness of the isolates from the city of Governador Valadares using genotypes at 4,728 polymorphic sites using PCA (**Figure 2A****)**, maximum likelihood (**Figure 2B****)** and population structure **(** **Figure 2C****)**. Results from these different approaches all point towards the presence of two well-defined clades of *L. (L.) infantum* within the city of Governador Valadares that correspond to two isolation periods, with all samples from 2008 and 2012 clustering together as a pre-2015 population, and samples from 2015 forming a separate group. The strength of the association between time of isolation and subpopulation was statistically significant by the Fisher exact test (p<0.001). We did not see any association with geographical location of the isolates within the city.

To test the relationship between this population and other isolates, phylogenetic analysis were carried out including data from other parts of Brazil (isolates MG12A, MA01A and PI04 from Montes Claros from Minas Gerais state, Paraibano from Maranhão state and Valença do Piauí from Piauí state, respectively) (Carnielli et al., 2018), three Old World *L. (L.) infantum* isolates from Israel (LRC-L1275), France (LEM1985) and Uzbekistan (LRC-L1311) (Franssen et al., 2020).

The best-fit model for the data according to the Akaike and Bayesian Information Criteria (AIC and BIC) and Decision Theory method (DT) was the GTR (generalised time reversible) substitution model with four rate categories. This model was used for maximum likelihood analysis and our results showed strong statistical support (with bootstrap values of 100%) for basal nodes of the tree, confirming a closer relationship among isolates from the city of Governador Valadares than to the Old World *L. (L.) infantum* isolates (**Figure 2B****)**.

### There Are Fixed SNPs in Coding Sequences That Differentiate Among Both Populations

Given the presence of two subpopulations, we sought to assess the presence and location of fixed SNPs in each group. We found 408 and 1,630 SNPs that were exclusively present in the 2008-2012 and 2015 subpopulations, respectively.

In the 2008-2012 subpopulation, there were 56 SNPs located in 26 genes that comprise hypothetical proteins, phosphatases and transport proteins. In the 2015 population there were 98 SNPs affecting 70 genes that include surface antigen proteins, kinesins and hypothetical proteins **(** **Supplementary Table 2****)**.

### The Governador Valadares Population Presents a Heterogeneous Pattern of Chromosomal and Gene Copy Number Amplifications on a Disomic Background

**Figure 3:**
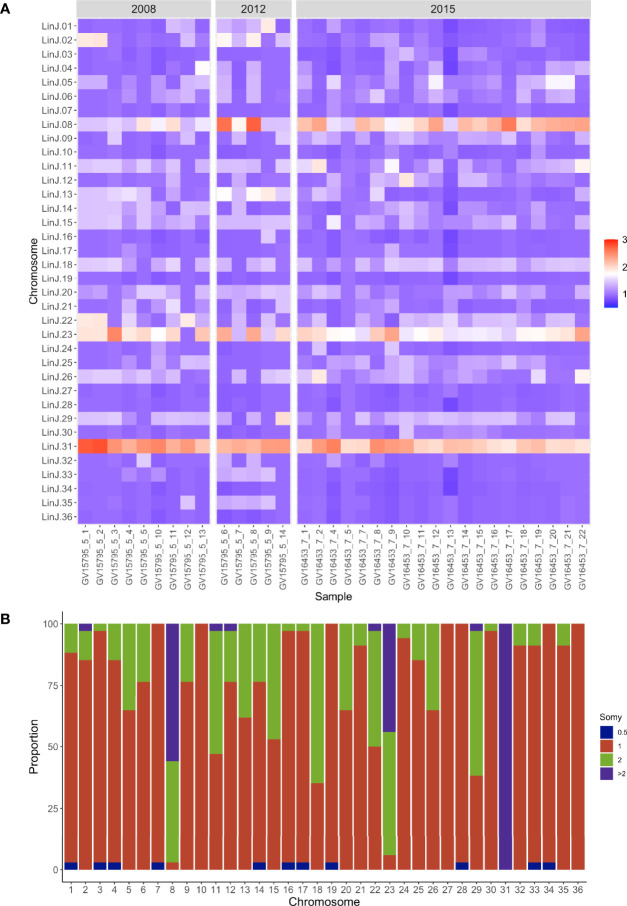
Aneuploidy in the Governador Valadares *L. (L.) infantum* isolates. **(A)** This heat map shows copy number of the 36 chromosomes, the y-axis indicates the chromosome and the x-axis the isolate. Colors indicate disomic (1, blue scale), trisomic (1.5, light blue scale), tetrasomic (2, light red scale) and pentasomic (2.5, red scale) chromosomes. The color key shows the normalized chromosome read depth and the frequency. **(B)** This graph shows the distribution of chromosome copy number for each chromosome in the population. The y-axis denotes the proportion of isolates and the x-axis the respective chromosome. The color key shows the normalized chromosome read depth and the frequency (0.5 monosomic; 1 disomic; 2 tetrasomic; >2 more than tetrasomic.

In *Leishmania*, the analysis of genome structure in single cells using fluorescence *in situ* hybridization (FISH) and next-generation sequencing have shown a wide range of variation in chromosomal content from cell to cell in a population resulting in intra-strain genomic heterogeneity (Sterkers et al., 2012) (Sterkers et al., 2011). This high variation has been referred to as mosaic aneuploidy and is proposed as a powerful adaptive mechanism in *Leishmania* to cope with the highly variable selective pressures in the vector and vertebrate hosts (Sterkers et al., 2014). Our data from the city of Governador Valadares show a highly heterogeneous pattern of ploidies in the population **(** **Figures 3A, B****).** In this analysis, only three out of 36 chromosomes were disomic in all the isolates from the city of Governador Valadares (10, 27, 36). Our results show that chromosome 31 has the highest dosage, being tetrasomic or pentasomic. This chromosome has been previously shown to be expanded in all *Leishmania* species so far sequenced and has been suggested to confer some selective advantage to the parasite (Valdivia et al., 2015). Other chromosomes that are present in more than two copies in most isolates include chromosome 8 and 23, whose ploidies range from tetrasomy to pentasomy **(** **Figures 3A, B****)**. Read depth on these chromosomes is uniformly distributed refuting region-specific amplification (data not shown). Also, no apparent somy variation across the two groups based on time of collection was observed.

Results from the CNV analyses were separated into expanded genes present in all the isolates tested and the ones expanded in each subpopulation. We found 75 genes with increased copy number in the 2015 group and 7 in the 2008-2012 group. In the case of the 2008-2012 group, 6 out of 7 differentially expanded genes were located on chromosome 31 whereas expanded genes in the 2015 isolates were located in chromosomes 8, 23 and 31. The majority of these genes were annotated as hypothetical proteins and kinases **(** **Supplementary Table 3****)**.

### Signatures of Selection on New World *L. (L.) infantum*

Quantifying how natural selection has affected the evolution of particular protein-coding genes allows a better comprehension of key players during the evolution of an organism. To assess natural selection on the *L. (L.) infantum* population of the city of Governador Valadares we employed a genome-wide scan of population genetic summary statistics in non-overlapping sliding windows of 10 kb. Although summary statistics indicates that the majority of the inferred SNPs are neutral (Tajima’s D = 0.433, Fu and Li’s F = 0.54), we were able to detect 34 chromosomal regions under balancing selection (**Supplementary Table 4**). These chromosomal regions include 88 genes that code mainly for hypothetical proteins and members of important antigenic families including surface antigen protein 2, proteophosphoglycan and amastins (**Supplementary Table 4**).

### Divergence Analysis Suggests Population Replacement Rather Than *In Situ* Divergence and Stabilization of the Effective Population Size

**Figure 4:**
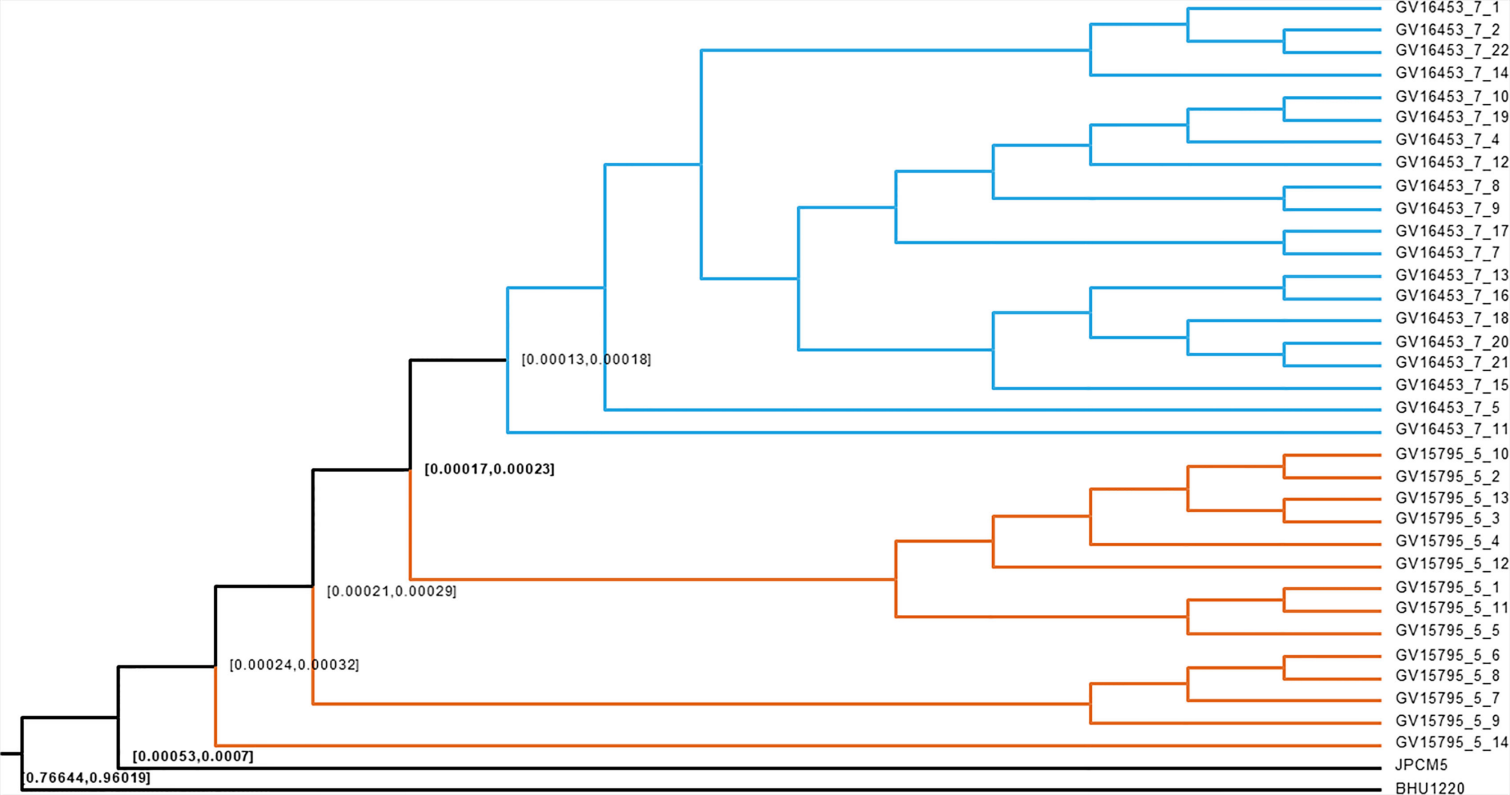
Bayesian dating of *L. (L.) infantum* isolates. The figure shows a cladogram corresponding to the strict clock model. Nodes are located at the mean divergence and numbers represent 95% confidence intervals. The result shows an earlier divergence of both *L. (L.) infantum* sub-populations in Governador Valadares. JPCM5: *L. (L.) infantum* JPCM5, BHU1220: *Leishmania (L.) donovani* BHU1220 strain. Colors in the branches distinguish the 2015 (Blue) and 2008/2012 (Orange) populations.

The age of divergence between the two Governador Valadares *L. (L.) infantum* subpopulations was inferred from 46,356 genotyped sites along the genomes and calibrated against the timing of the split between Old World and New World *L. (L.) infantum*. Bayesian based divergence time analysis using the strict and relaxed clock models resulted in fairly similar dates, which point towards a recent divergence of both populations after the introduction of *L. (L.) infantum* into the New World. The 95% highest posterior intervals for the most recent common ancestor between both populations was 490-300 years ago according to the relaxed clock model and 231-171 for the strict clock model, **(** **Figure 4****)**. These results confirm that the changes in the *L. (L.) infantum* genotypes circulating in the city of Governador Valadares are due to population replacement rather than recent *in-situ* divergence.

### Linkage Disequilibrium Analysis Suggests That Neither of the Two *Leishmania* Subpopulations Are Clonal

**Figure 5:**
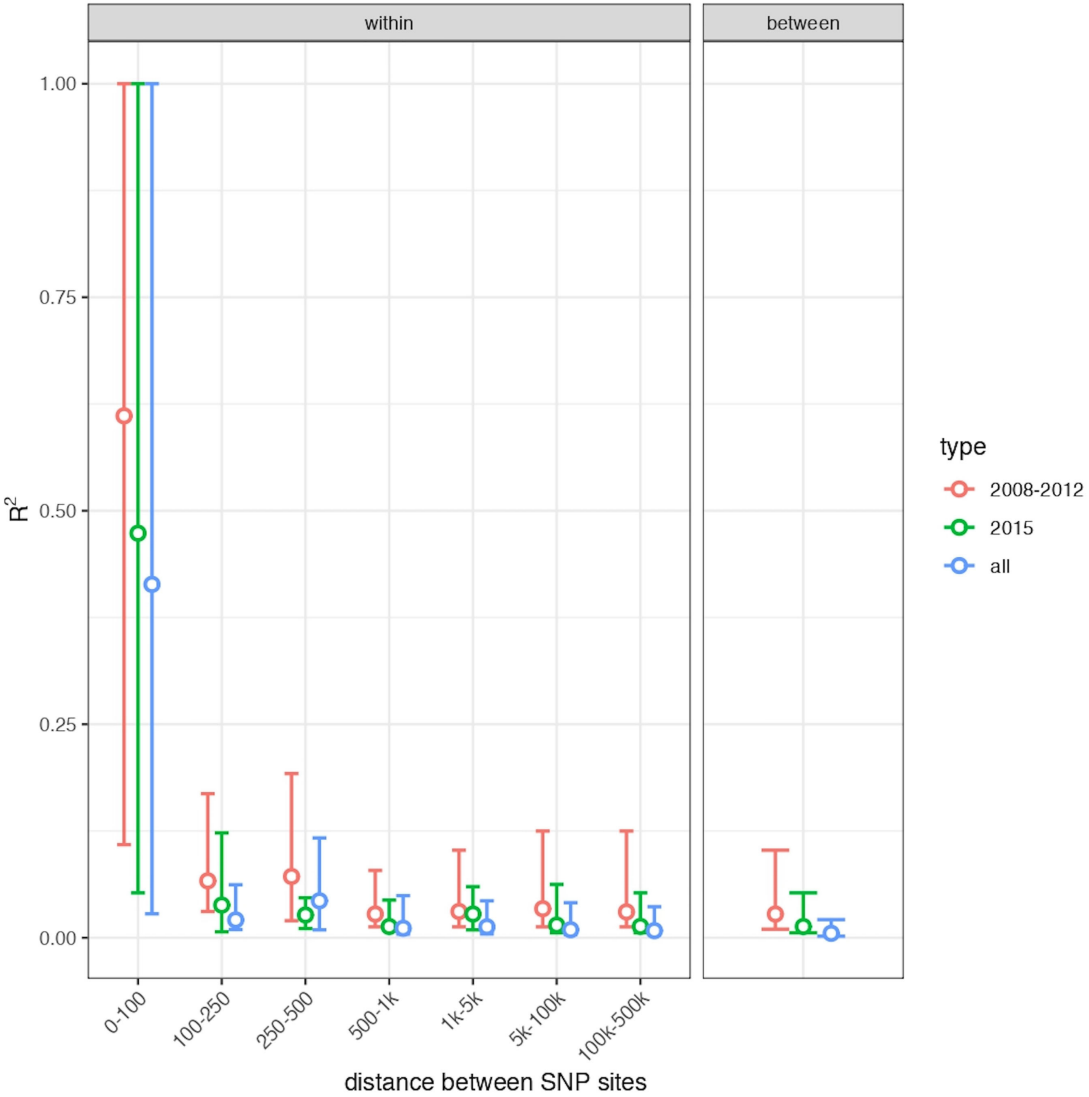
Linkage disequilibrium decay with genomic distance. Points show the median and bars show the interquartile range of R^2^ for all pairs of SNP variants within each category of physical distance on the genome for SNPs on the same chromosome (left panel) and for all pairs of SNPs on different chromosomes (right panel). Values are shown separately for Governador Valadares samples from 2008/2012, Governador Valadares samples from 2015 and both groups together, in each case including only SNPs that vary within the relevant group of samples. The absolute values of R^2^ are affected by the number of samples in a group, but all sets show a similar, rapid decay in linkage disequilibrium, suggesting frequent recombination within each group.

To investigate the occurrence of recombination within each 2008/2012 and 2015 subpopulations, the decay in linkage between SNPs as a function of their physical distance was measured. As shown in **Figure 5**, a rapid decay of R^2^ with genomic distance was observed, supporting the conclusion that neither of these populations are clonal, but rather reveals high rates of sexual recombination within each population. Altogether, the divergence and linkage disequilibrium analyses indicate that, within a two-year period, one sexual population was replaced by the other one, which was originated from elsewhere.

### Climate Data Shows a Significant Change in Precipitation During 2014

Analysis of daily temperature between 2008 and 2014 shows that temperature in the city

Governador Valadares presents two distinct phases along the year **(** **Supplementary Figure 2****)**. There is one colder season that starts in May and ends in August and a warmer season from September to April. However, we were not able to find any statistically significant difference in temperature cycles during these years.

**Figure 6:**
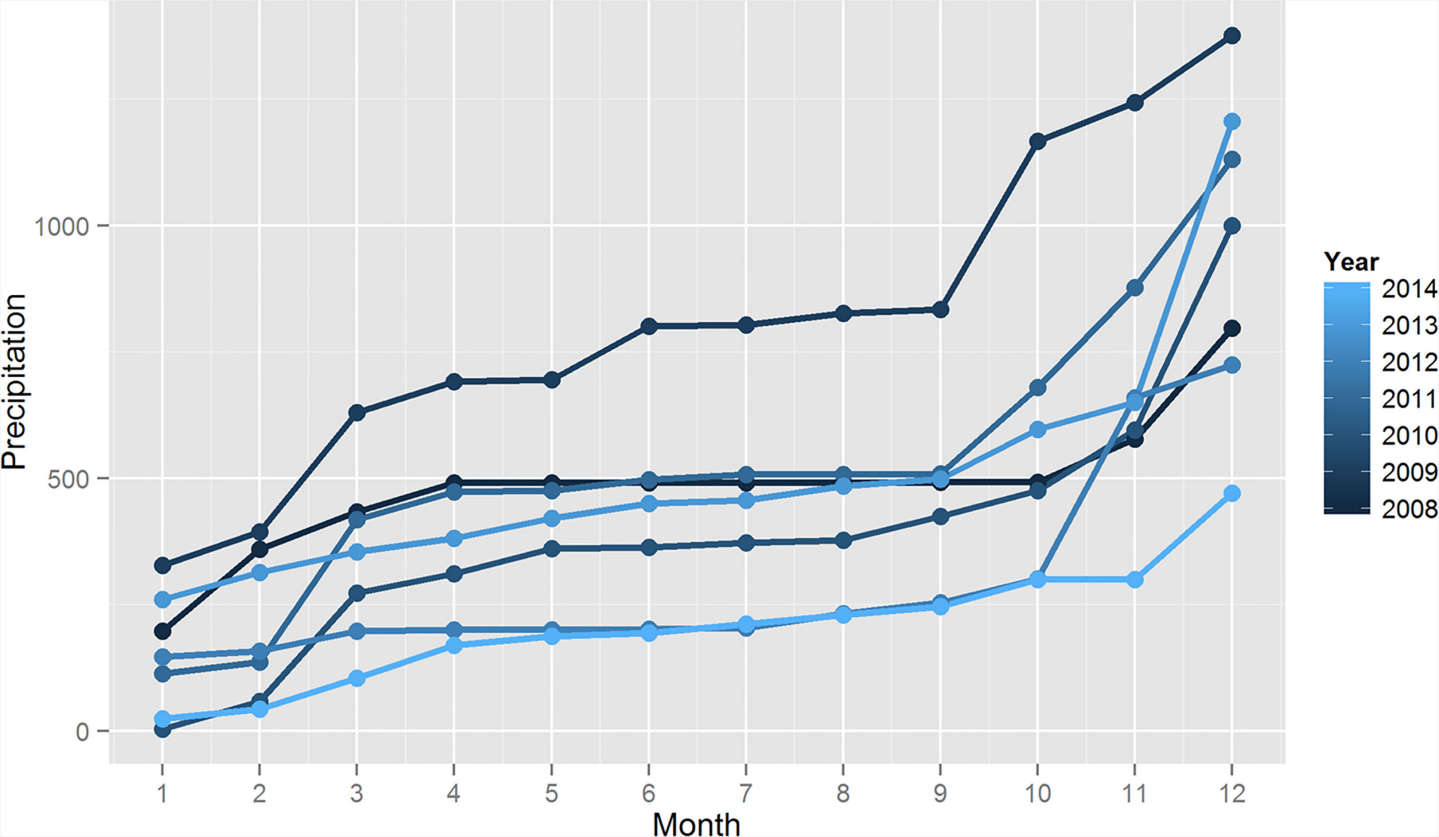
Precipitation data in the city of Governador Valadares. Inset shows cumulative precipitation in Governador Valadares since 2008 to 2014. Analysis of the data suggest a significant decrease in rainfall during 2014.

We also found that precipitation appears to be cyclic with a rainy season that starts in September and move up to March. Annual precipitation rates suggest a decrease in total rainfall in 2014 **(** **Figure 6****)**. To assess if this difference in total rainfall is significant, we compared annual precipitations since 2008 up to 2014 using the Worsley likelihood ratio test implemented in TREND. The result of this analysis shows a statistically significant result (p<0.05) for changes in the mean precipitation of 2014 compared to the other years evaluated.

## Discussion

Leishmaniasis is a major health problem in endemic countries in tropical and sub-tropical areas and has been linked to poverty and limited access to health care. Control of these diseases has been difficult to achieve as evidenced by outbreaks in several countries and the increase of endemic regions (Silveira et al., 2002; Velez et al., 2012; Arce et al., 2013; Uranw et al., 2013). Among the leishmaniasis, VL is considered as the most aggressive form of these diseases and can be deadly if untreated (Murray, 2004; Murray et al., 2005). In the New World, Brazil is the country that holds most VL cases (Alvar et al., 2012) and according to WHO it is among the ten countries that hold more than 90% of all VL cases in the world.

Currently there is increased concern about the effects that climate change, human activities and co-infections might have on the leishmaniasis. For instance, migration and urbanization with their implicit effects (deforestation, settlement near the forest and breeding of animals) have led to an increase of TL and VL cases and have posed a challenge for control programs in different regions. This increase in cases is reinforced by an expansion in the distribution of sand fly vectors, pathogenic *Leishmania* species and the re-emergence of infections in previously controlled areas (Arce et al., 2013; Barata et al., 2013; Desjeux, 2004). Improved knowledge of the population structure of pathogens can provide insights into transmission patterns and other epidemiological features and can be a valuable ally in the study of parasite population emergent and re-emergent foci of leishmaniasis.

The region of Governador Valadares is a re-emergent focus of VL with both, *L (L.) infantum* and *L. (L.) amazonensis* species circulating in the area despite intense control efforts. Our analyses have revealed the presence of two distinct sexual subpopulations of *L. (L.) infantum* in this focus that are associated with date of isolation and that apparently diverged hundreds of years ago. This excludes the possibility that these clades diverged during the period of time that no isolates were taken and suggests a shift in the *L. (L.) infantum* population in which the predominant population in this focus became replaced by a distinct parasite population within a two-year period.

In the absence of conclusive evidence, we can only speculate as to a possible cause of this event. The replacement could be associated with a change in precipitation during 2014 that may have had an impact on the sand fly population in the area, and in turn could have affected the circulating parasite populations. It is important to emphasize, however, that additional studies are necessary to investigate whether climate changes drove the subpopulation replacement observed here. It is unclear whether the more recent population was always present in this focus, at a low enough frequency that our sampling did not detect these parasites during the earlier timeframe, or whether these parasites were recently introduced into the city from elsewhere.

Previous evidence from a spatial-temporal analysis in French Guiana showed a negative correlation between rainfall and the number of TL infections. This finding suggests that rainfall could be an indicator for risk in endemic sites with an increase in the number of cases of *Leishmania* infections after 2 months of relative decrease in rainfall (Roger et al., 2013).

Chromosome and gene copy number variations are a major source of intra and interspecific variability in *Leishmania* (Rogers et al., 2011; Sterkers et al., 2012; Valdivia et al., 2015) and have been suggested to be an adaptation that favors parasitism by modulating expression levels through changing gene dosage (Iantorno et al., 2017; Bussotti et al., 2018). The Governador Valadares population presents a highly diverse pattern of ploidies with extensive variability among isolates regardless of their clustering in the two subpopulations previously described and the very recent divergence inside each subpopulation.

Signatures of selection on surface proteins such as amastins observed in our study highlight the continuing evolution of *Leishmania* as previous research has showed that gene gain of this and other multigene families had a significant role on the evolution of parasitism in this species (Jackson et al., 2016). Furthermore, the presence of species/specific clusters of surface proteins such as amastins, PSA, GP63 are indicative of adaptations at the species level on proteins involved in host parasite interactions. In addition, the presence of selection signatures on proteophosphoglycans is likely a reflect of the interaction of the parasite with the insect vector as it has been shown that these proteins have an important role during stage specific development within the insect vector (Coutinho-Abreu et al., 2020).

The rapid replacement of *L. infantum* population in the city of Governador Valadares is likely a contributing factor that challenge visceral leishmaniasis control in this endemic focus. This study has increased our understanding of the *Leishmania* population structure in the city of Governador Valadares that deserve to be explored in other endemic sites in the New World. The importance and complexity of the leishmaniasis herein shown demand the use of genomic approaches to understand disease transmission and develop efficient intervention strategies. The information that can be provided by these endeavors could contribute to the design of better control strategies, especially in the New World.

## Data Availability Statement

The datasets presented in this study can be found in online repositories. The names of the repository/repositories and accession number(s) can be found in the article/**Supplementary Material**.

## Ethics Statement

The animal study was reviewed and approved by Committees of Ethics in Animal Experimentation of the Universidade Federal de Ouro Preto (protocol number 083/2007). Written informed consent for participation was not obtained from the owners because According to the Brazilian Ministry of Health, VL-seropositive dogs must be euthanized as a measure to control VL expansion in the country. Two independent serological tests must be positive to characterize a positive animal: the Dual-Path Platform Immunochromatographic Rapid Test (TR-DPP) as the screening method and the enzyme-linked immunoassay (ELISA EIE) as the confirmatory test. The Leishmania infantum parasites used in this study were obtained from dogs reactive in both tests and after they were euthanized in the Governador Valadares Zoonose Control Center under agreement no 01819.

## Author Contributions

HV carried out the bioinformatics analyses, participated in study conception and design and drafted the manuscript; JO and RB carried out bioinformatics analyses and drafted the manuscript; BR and AR contributed to the study conception and design; MS coordinated the genome sequencing; JC participated in the study design, bioinformatics analyses, coordination, and writing of the manuscript. DB participated in the study concept and design, bioinformatics analysis, coordination and writing of the manuscript. All authors contributed to the article and approved the submitted version.

## Funding

DB group is supported by Minas Gerais State Agency for Research and Development (FAPEMIG), the Brazilian Federal Agency for Support of Graduate Education (CAPES), and Brazilian Council for Scientific and Technological Development (CNPq). DB and AR are CNPq research fellows. JO and AC-D-S received scholarship from CAPES and CNPq, respectively. This work as funded in part by the Wellcome Trust [grant number 206194]. For the purpose of Open Access, the author has applied a CC BY public copyright licence to any Author Accepted Manuscript version arising from this submission.

## Conflict of Interest

The authors declare that the research was conducted in the absence of any commercial or financial relationships that could be construed as a potential conflict of interest.

## Publisher’s Note

All claims expressed in this article are solely those of the authors and do not necessarily represent those of their affiliated organizations, or those of the publisher, the editors and the reviewers. Any product that may be evaluated in this article, or claim that may be made by its manufacturer, is not guaranteed or endorsed by the publisher.

## Supplementary Material

Supplementary Figure 1 The figure shows the estimated seroprevalence of *L. (L.) infantum* in the canine population of Governador Valadares. Data corresponds to active surveillance activities conducted since 2008 up to 2014 by VL control program of Governador Valadares.

Supplementary Figure 2 Heatmap of mean monthly temperatures since 2008 to 2014 in Governador Valadares showing the presence of two clear cycles of temperature. There is a warmer period that starts in September and finishes in the end of April of the following year. A second colder period starts in May and finishes in August of the same year.
