# Supplementary figures and images for: Replacement of Leishmania (Leishmania) infantum Populations in an Endemic Focus of Visceral Leishmaniasis in Brazil
Source: Front Cell Infect Microbiol. 2022 Jun 24;12:900084. doi: 10.3389/fcimb.2022.900084 (PMC9263273; doi:10.3389/fcimb.2022.900084)

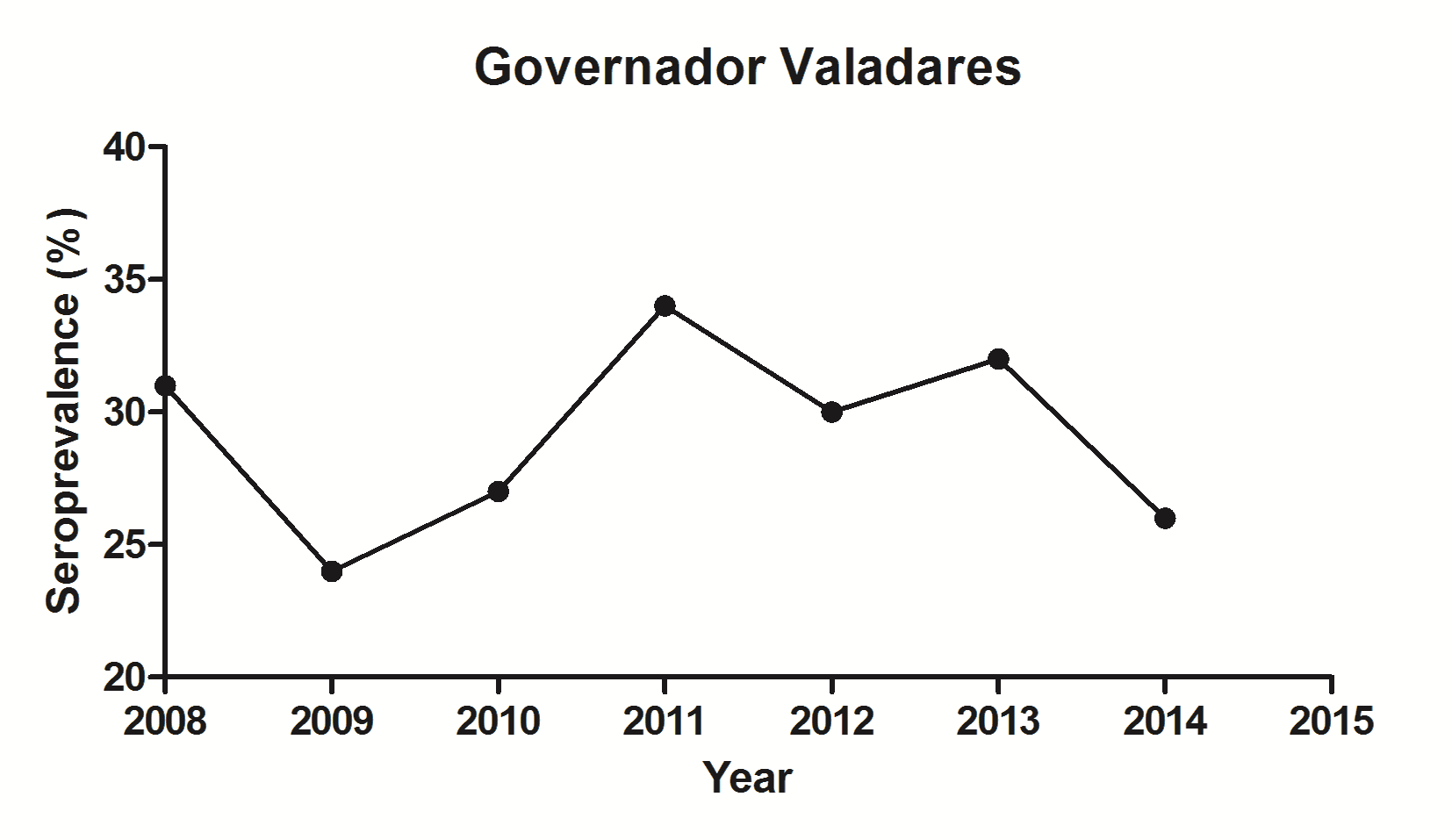

Supplement: Supplementary Figure 1 — The figure shows the estimated seroprevalence of L. (L.) infantum in the canine population of Governador Valadares. Data corresponds to active surveillance activities conducted since 2008 up to 2014 by VL control program of Governador Valadares. [file Image_1.tiff]

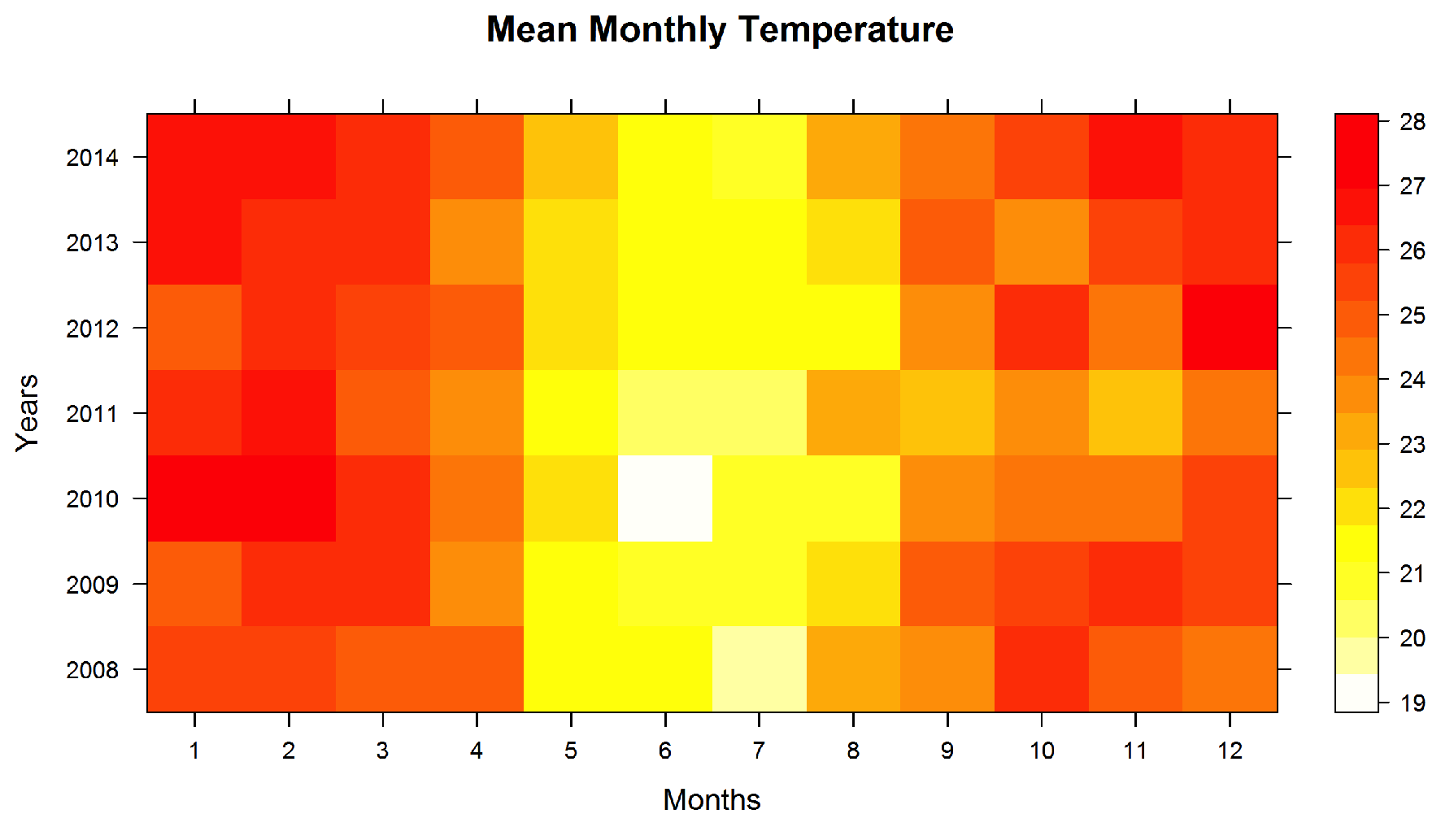

Supplement: Supplementary Figure 2 — Heatmap of mean monthly temperatures since 2008 to 2014 in Governador Valadares showing the presence of two clear cycles of temperature. There is a warmer period that starts in September and finishes in the end of April of the following year. A second colder period starts in May and finishes in August of the same year. [file Image_2.png]
